# The adverse drug reactions to tumor necrosis factor alpha inhibitor

**DOI:** 10.1186/2045-7022-4-S3-P53

**Published:** 2014-07-18

**Authors:** Hye-Kyung Park, Eun-Jung Jo, Seung-Eun Lee, Jung-Ha Mok, Mi-Hyun Kim, Kwangha Lee, Ki-Uk Kim, Min-Ki Lee

**Affiliations:** 1 Pusan National University School of Medicine, Department of Internal Medicine, Korea, Republic of; 2 Pusan National University Yangsan Hospital, Department of Internal Medicine, Korea, Republic of

## Background

Biologic therapies targeting tumor necrosis factor alpha (TNFa) are a mainstay in the treatment of autoimmune diseases such as ankylosing spondylitis (AS), rheumatoid arthritis (RA), or Crohn's disease (CD). With increased use of the biologic therapies, various adverse effects to TNFa inhibitor have been reported.

## Method

We reviewed retrospectively the clinical data of subject who were treated with infliximab, adalimumab, or etanercept from 2006 to 2012, and assessed the adverse drug reactions using electronic medical recording system in Pusan National University Hospital.

## Results

In total, 111 subjects were enrolled. Mean age was 39.8¡¾11.7 years, and male was 68.5%. The diagnoses of subjects were AS (62.2%), RA (24%), CD (17%), and pyoderma gangrenosum (0.9%). The injected agents were adalimumab (53.2%), etanercept (29%), and infliximab (20.7%). Adverse drug reactions of 30.6% were reported. The most common adverse reactions were cutaneous reactions; 10 (9.0%) eczema, 4 (3.6%) injection site reaction, and 2 (1.8%) urticaria. 3.6% of subjects had a slight increase in liver enzyme, and pulmonary tuberculosis occurred in 2 (1.8%) patients. Mean therapy duration prior to adverse reactions was 2.1¡¾2.2 years. Serious adverse reaction, anaphylaxis, growth retardation, and Henoch-Schönlein purpura occurred in one patient each.

## Conclusion

This study shows that the adverse reactions to TNFa inhibitors were frequent, but most of them were mild reactions and the most common adverse reaction was cutaneous reactions.

